# Hollow WO_3_/SnO_2_ Hetero-Nanofibers: Controlled Synthesis and High Efficiency of Acetone Vapor Detection

**DOI:** 10.3389/fchem.2019.00785

**Published:** 2019-11-19

**Authors:** Hongyun Shao, Minxuan Huang, Hao Fu, Shaopeng Wang, Liwei Wang, Jie Lu, Yinghui Wang, Kefu Yu

**Affiliations:** ^1^School of Marine Sciences, Guangxi University, Nanning, China; ^2^Guangxi Laboratory on the Study of Coral Reefs in the South China Sea, Nanning, China; ^3^Guangxi Key Laboratory of Processing for Nonferrous Metallic and Featured Materials, Nanning, China; ^4^College of Life Science and Technology, Guangxi University, Nanning, China

**Keywords:** electrostatic spinning, hollow nanofiber, WO_3_/SnO_2_ heterojunction, acetone, gas sensor

## Abstract

Metal oxide hetero-nanostructures have widely been used as the core part of chemical gas sensors. To improve the dispersion state of each constituent and the poor stability that exists in heterogeneous gas sensing materials, a uniaxial electro-spinning method combined with calcination was applied to synthesize pure SnO_2_ and three groups of WO_3_/SnO_2_ (WO_3_ of 0.1, 0.3, 0.9 wt%) hetero-nanofibers (HNFs) in our work. A series of characterizations prove that the products present hollow and fibrous structures composed of even nanoparticles while WO_3_ is uniformly distributed into the SnO_2_ matrix. Gas sensing tests display that the WO_3_/SnO_2_ (0.3 wt%) sensor not only exhibits the highest response (30.28) and excellent selectivity to acetone vapor at the lower detection temperature (170°C), 6 times higher than that of pure SnO_2_ (5.2), but still achieves a considerable response (4.7) when the acetone concentration is down to 100 ppb with the corresponding response/recovery times of 50/200 s, respectively. Such structure obviously enhances the gas sensing performance toward acetone which guides the construction of a highly sensitive acetone sensor. Meanwhile, the enhancement mechanism of such a special sensor is also discussed in detail.

**Graphical Abstract:**
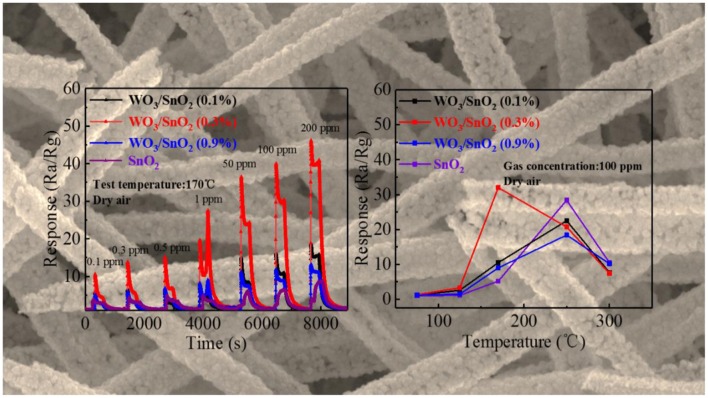
Response curves or hollow WO_3_/SnO_2_ hetero-nanofibers at different concentrations and temperatures.

## Introduction

The air quality in the workplace and living environment is closely related to people's health, it is therefore urgent to detect air pollution quickly and accurately. Acetone, a common organic volatile solvent, is widely used in laboratories and industries, posing a threat to the nose, eyes, and central nervous system of the human body when the concentration in the environment reaches 0.90 ppm (Hygienic Standard for Design of Industrial Enterprises) (Yang C. et al., 2019). Additionally, clinical data indicate that certain components of exhaust gases can be regarded as diagnostic markers of certain diseases, to that end acetone can be used as a marker of diabetes (Parthibavarman et al., 2018). Therefore, chemical gas sensors have emerged as many advantages such as portable, real-time and online detection are required (Meng et al., 2017; Yuan et al., 2019). Thus the development of acetone gas sensor can supply better service for safety control and human health (Meng et al., 2019).

To date, a great deal of research still focuses on fabricating high performance metal oxide semiconductor (MOS) gas sensing materials due to their many irreplaceable advantages such as their stability and their reliable sensing (Kucheyev et al., 2006; Das and Jayaraman, 2014; Yang and Guo, 2016; Teng et al., 2019), especially in improving the gas sensing properties of SnO_2_-based nanomaterials through the assembly into heterojunctions (Das and Jayaraman, 2014; Koohestani, 2019; Teng et al., 2019). It is known that pristine SnO_2_-based sensors often work at higher temperatures (>300°C) with poor selectivity, a lack of reproducibility, and an inadequate detection limit, thereby restricting their practical application (Wang et al., 2016; Wang L. et al., 2019). Hetero-junction constructing has become attractive since it produces intimate interface-contact directly between two different semiconductor materials, thus balancing the Fermi level, forming the thicker depletion layer on the interface, and finally increasing the sensor performances (Wang et al., 2016; Zhao et al., 2019).

Furthermore, SnO_2_ composed of a hollow/porous nanostructure can help increase the surface area remarkably, and provide more channels to transfer electrons (Lu et al., 2011). 1D nanofibers (NFs) possess a high surface area-to-volume ratio, excellent stability, and can easily be modified which are favorable for enhancing response signals (Xia et al., 2010), while WO_3_ has been widely studied in gas sensors with advantages of low temperature, high sensitivity, and low detection limit (Joshi et al., 2018; Li et al., 2018; Sukunta et al., 2018). By combining the advantages of 1D and hollow/porous structures, more attention is now being focused on the construction of the WO_3_/SnO_2_ heterojunction. In comparison with other synthetic methods, like the hydrothermal or impregnation method, electro-spinning is the most effective method to uniformly control one-dimensional hetero-nanofibers (HNFs) with a high yield and favorable stability (Ma et al., 2010; Wang et al., 2013).

Inspired by the newly reported 1D HNFs and with the intention of artificially tuning the gas-sensing performances, electro-spinning was used to construct hollow WO_3_/SnO_2_ HNFs with different weight ratios of WO_3_ (Patil et al., 2017; Wang K. et al., 2019). Herein, the synthesized sensor based on WO_3_/SnO_2_ (0.3 wt%) could be a good candidate for acetone pollutant monitoring. Its gas sensing properties and working mechanism in particular will be evaluated and discussed systematically.

## Experimental Section

### Synthesis of Hollow WO_3_/SnO_2_ HNFs

All the reagents used in the experiment, including Tin(II) choride dehydrate (SnCl_2_·2H_2_O, AR, ≥98%), N,N-dimethylformamide (DMF, AR, 99.5%) and polyvinylpyrrolidone (PVP, K = 1300000, K88-96) were purchased from Aladdin Industrial Corporation (Shanghai, China). Sinopharm Chemical Reagent Co. Ltd. (Shanghai, China) provides ammonium tungstate hydrate ((NH_4_)_10_W_12_O_41_·xH_2_O, AR) and ethanol (CH_3_CH_2_OH, AR). Furthermore, distilled water was used in the synthesis procedures.

An electrostatic spinning method was applied for the preparation. Typically, 1 mmol of SnCl_2_·2H_2_O and a certain amount of (NH_4_)_10_ W_12_O_41_·xH_2_O was dissolved in 2.32 ml of DMF and 2.79 ml of anhydrous ethanol under stirring. Then, 0.0003 mmol of PVP (K = 1300000) was added before the precursor solution was prepared. By controlling the amount of (NH_4_)_10_W_12_O_41_·xH_2_O, we designed different mass percentages of WO_3_ as 0, 0.1, 0.3, 0.9 wt% respectively in the WO_3_/SnO_2_ products, which were denoted as pure SnO_2_, WO_3_/SnO_2_ (0.1%), WO_3_/SnO_2_ (0.3%), and WO_3_/SnO_2_ (0.9%). Then the configured solution was transferred in the 5 ml syringe, then 20–22 kV of positive voltage was added to the needle and 3–4 kV of negative voltage to the receiver of the sample. The distance between the needles and the receiver was 15 cm, and the jet velocity of the fluid from the syringe was kept 2.32 mL of DMF and 2.79 mL as 0.2 mm/min under a high voltage electric field. In the whole electrostatic spinning process, the temperature was kept at 60°C and humidity of 10%. Afterwards, the white film was collected and calcined at 600°C, for 2 h in the air state, and finally different HNFs products were obtained.

### Characterization of Hollow WO_3_/SnO_2_ HNFs

The samples were characterized by means of powder XRD analysis (Rigaku Ultima IV, Japan; Cu Kα radiation, λ = 1.5418 Å), Field-emission SEM (Hitachi SU5000, Japan), TEM and HRTEM with EDS (FEI Tecnai G2 f20 s-twin, 200 KV), XPS (Thermo SCIENTIFIC ESCALAB 250Xi, Al Ka X-ray monochromator), BET (Autosorb-IQ, USA).

### Preparation of Gas Sensor and Sensing Test

The detailed manufacturing process of gas sensors can be found in our previous reports (Wang L. et al., 2019). Briefly, the sample was mixed with deionized water and ground until it became sticky and was then coated onto the surface of the ceramic tube, which was welded to the pedestal and inserted into the test channel. A gas-sensing test was carried out using the CGS-8 intelligent test system (Beijing Elite Tech Co. Ltd., China). The response value (S) of sensors based on n-type semiconductor is defined as S = R_a_/R_g_, where R_a_ and R_g_ represent the resistance value of the sensor in air and in target gas, respectively, while the p type is the opposite. Response time is defined as the time taken by the sensor to reach 90% of the final equilibrium value, or the recovery time for gas desorption. Most tests were carried out under dry air conditions, except the discussion of RHs effect on the response of different sensors (Xia et al., 2010; Wang et al., 2013; Wang L. et al., 2019).

## Results and Discussion

### Characterization

**Figure 1:**
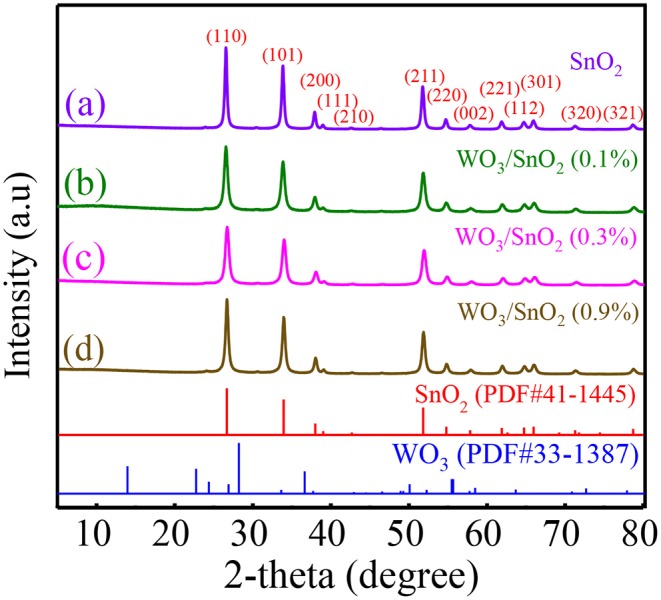
XRD patterns of **(a)** pure SnO_2_, and **(b)** WO_3_/SnO_2_ (0.1%), **(c)** WO_3_/SnO_2_ (0.3%), and **(d)** WO_3_/SnO_2_ (0.9%) HNFs.

X-ray diffraction was utilized to confirm the crystal structure of as-prepared samples. As shown in Figure 1a, the sharp diffraction peaks represent the pure tetragonal SnO_2_ phase (JCPDS file No. 41-1445) with high crystallinity and large crystal size. Additionally, the main diffraction patterns in Figures 1b–d can also be indexed to tetragonal SnO_2_, but no obvious WO_3_ peak presents due to its low content or highly dispersed amorphous state in the HNFs. Then the grain sizes of WO_3_/SnO_2_ (0.1%, 0.3%, 0.9%) and pure SnO_2_ samples were calculated by the Scherrer formula taken from 2θ = 26.6° of the XRD data, which were 17.53, 15.71, 20.56, and 22.70 nm, respectively. It means that the grain size was smallest in the sample of WO_3_/SnO_2_ (0.3%) HNFs. It is overwhelmingly known that when crystallite sizes approach Debye length (λ_D_, usually several nm), the sensor response will drastically increase, and the smaller the better (Miller et al., 2014; Nan et al., 2019). The conclusion can therefore be drawn from the XRD results that the WO_3_/SnO_2_ (0.3%) sample with the smallest grain size, 15.71 nm, would exhibit excellent gas sensing performance in the following tests, and all the other characterizations were taken based on this example.

**Figure 2:**
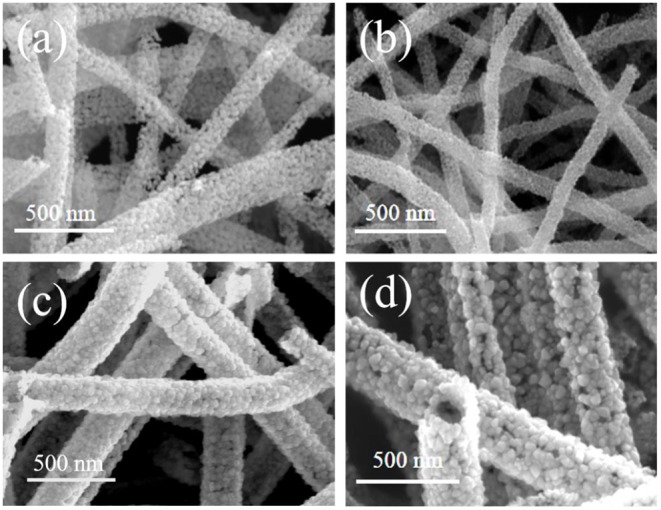
SEM images of the **(a)** pure SnO_2_, and the **(b)** WO_3_/SnO_2_ (0.1%), **(c)** WO_3_/SnO_2_ (0.3%), and **(d)** WO_3_/SnO_2_ (0.9%) HNF samples, respectively.

**Figure 3:**
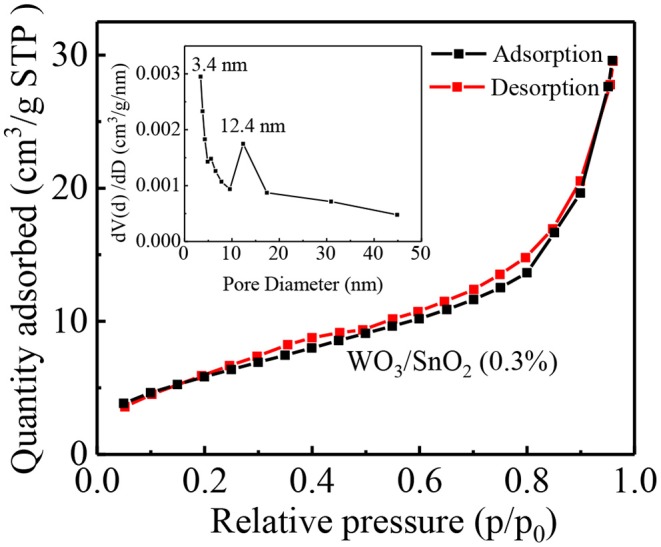
N_2_ adsorption-desorption isotherm with the BJH pore-size distribution of WO_3_/SnO_2_ (0.3%) HNFs.

The morphology of each sample, namely the pure SnO_2_, and WO_3_/SnO_2_ (0.1, 0.3, and 0.9%) HNF samples, respectively, were investigated by FESEM. As can be seen in Figure 2a, a uniform and hollow SnO_2_ NF is composed of large numbers of nanoparticles with an average diameter of ~18 nm, which is derived from the electro-spinning and heat treatment. Regarding the WO_3_/SnO_2_ HNF samples in Figures 2b–d, they show similar morphologies to that of the pristine SnO_2_ NF (Figure 2a), but as the amount of WO_3_ increases, the average diameters of the particle-like construction units increase gradually to 22, 48, and 52 nm respectively. SEM results showed that the surface structure of SnO_2_ NF had been changed by the doping of WO_3_, which may indirectly prove the existence of WO_3_. Such results are also proven through other investigations (HRTEM and XPS) in the following sections. Since the WO_3_/SnO_2_ composite is comprised of aggregated nanoparticles with porous structures, the N_2_ adsorption–desorption method (Figure 3) was applied on WO_3_/SnO_2_ (0.3%) to confirm the speculation, as can be calculated from the data in Figure 3 which shows that the specific surface area is 22.05 m^2^/g with a pore size diameter in the range of 3.4–45 nm averaged at 12.4 nm, proving its mesoporous structure for the composite. Therefore, the porosity of the sensing materials could improve its sensitivity due to the high surface area and rapid gas diffusion.

**Figure 4:**
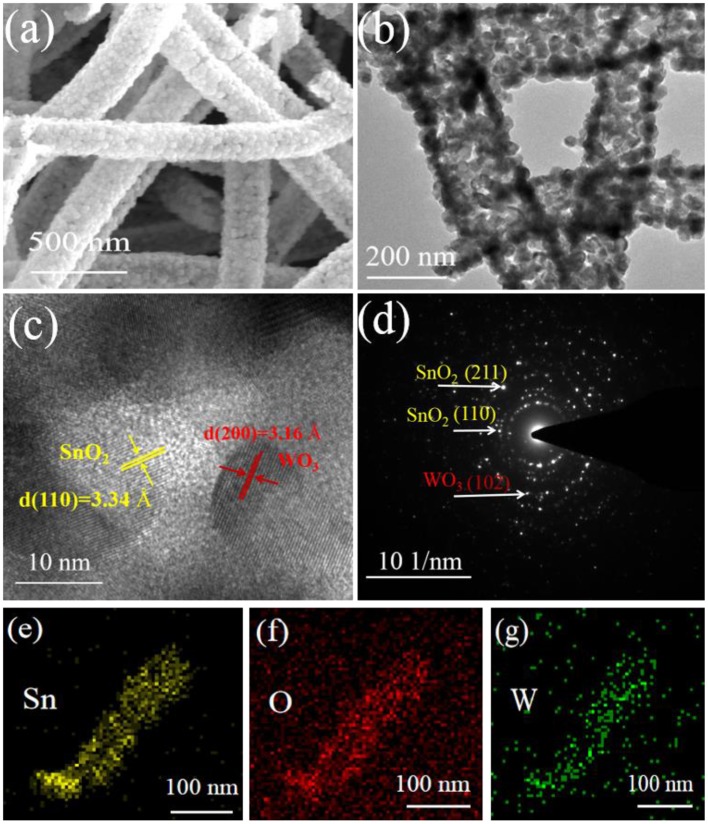
**(a)** SEM image, **(b)** TEM image, **(c)** HRTEM image and **(d)** SAED of WO_3_/SnO_2_ (0.3%) sample, with the **(e–g)** elemental mappings of Sn, O, and W elements.

To more clearly provide insight into the microstructure, TEM & HRTEM were taken on the WO_3_/SnO_2_ (0.3%) HNFs. Corresponding to the SEM image in Figure 4a, the sample in the enlarged (Figure 4b) presents a hollow structure composed of various crossly-dispersed and stereoscopic nanofibers with width of ~200 nm. In addition, it can be seen in Figure 4c that the interplanar spacings of the SnO_2_ (110) and WO_3_ (200) are 0.334 and 0.316 nm, respectively. The SAED pattern (Figure 4d) displays the polycrystal characteristic of the composite, while the elemental mapping shown in Figures 4e–g proves the existence of Sn, O and W elements in the final composite; in particular, W is uniformly distributed along the SnO_2_ matrix. The corresponding EDS spectrum image and content of Sn, O and W elements were exhibited in Figure S1.

**Figure 5:**
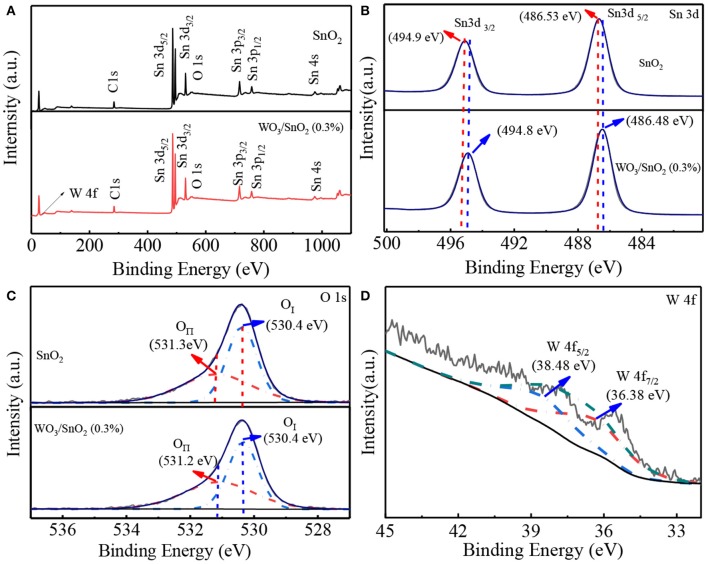
**(A)** Survey spectra, and high magnification XPS spectra of **(B)** Sn 3d, **(C)** O1s based on pure SnO_2_ and WO_3_/SnO_2_ (0.3%) HNF samples, as well as **(D)** W 4f in WO_3_/SnO_2_ (0.3%) HNF, respectively.

As is known, combining of the W element should bring interface change that then affects the chemical state of the SnO_2_ substrate (Tang et al., 2014; Stojadinović et al., 2016). In order to obtain a clearer vision the elemental chemical status of pure SnO_2_ and WO_3_/SnO_2_ (0.9%) HNFs were confirmed by XPS in Figure 5. Besides the peaks of Sn, O and C, a tiny peak of W 4f can be seen in the XPS spectra of the WO_3_/SnO_2_ (0.3%) composite (Figures 5A,D), consistent with the results shown in Figure 4d. The binding energy of Sn 3d_3/2_ (494.98 eV) and Sn 3d_5/2_ (486.58 eV) in particular, are displayed in the pure SnO_2_, but a minor negative shift of 0.18 eV can be noted for the Sn 3d_3/2_ (494.8 eV) and Sn 3d_5/2_ (486.4 eV) in the WO_3_/SnO_2_ (0.3%) composite (Figure 5B) (Lavacchi et al., 2000; Liu et al., 2016). Furthermore, a minor negative shift of the binding energy is also observed for the element of O 1s (Figure 5C), i.e., the lattice oxygen [530.38 eV (O_I_)] and chemisorbed oxygen (531.24 eV (O_II_)) for pure SnO_2_, while 530.38 eV (O _I_) and 531.11 eV (O_II_) are the values for the WO_3_/SnO_2_ (0.3%) composite, wherein the chemisorbed oxygen of O_II_ shifts negatively for 0.13 eV (Yang and Guo, 2016; Yang C. et al., 2019). Note that the gas sensing property is quite relative to the content of chemisorbed oxygen which increases by 9% after W modification, so the sensing performance would be remarkably improved (Teng et al., 2019). Finally, in Figure 5D, W 4f in the WO_3_/SnO_2_ (0.3%) composite gives a spin orbital dipole with two binding energies of 36.38 and 38.48 eV, respectively. The calculation results show that the spin orbit jet energy is 2.1 eV, which reaches a similar agreement with the theoretical calculation value (Nayak et al., 2015; Bai et al., 2016; Tofighi et al., 2019). According to the XPS results, interaction with the decoration of WO_3_ is clearly witnessed, leading to the modification of the chemical state of the WO_3_/SnO_2_ (0.3%) composite. In particular, the improved gas properties will be compared with pure SnO_2_ and discussed in detail in the gas sensitivity test section.

### Gas Sensing Test

**Figure 6:**
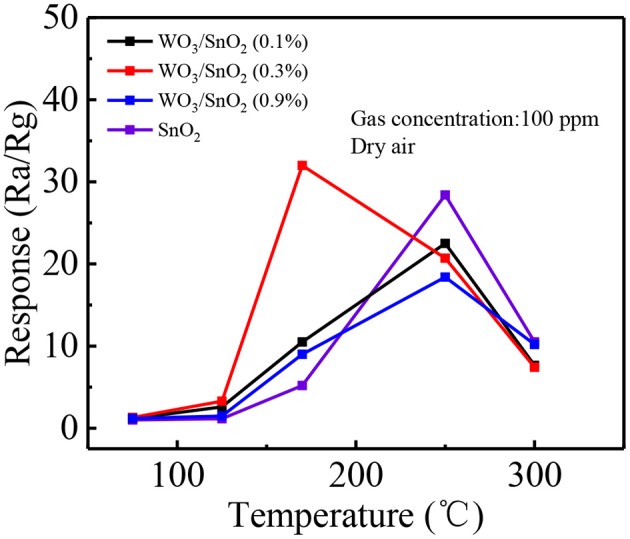
Response of the sensors based on above sensing materials to 100 ppm of acetone at operating temperatures from 50 to 300°C.

As mentioned, the WO_3_/SnO_2_ (0.3%) HNF could be a good gas sensor candidate, since its hollow channel and porous structure may accelerate the gas diffusion and reaction on the active sites. Moreover, the interaction of W nanoparticles with the SnO_2_ substrate can promote the O_2_ chemisorption and generate a higher response when exposed to an acetone pollutant at low concentrations. Consequently, the optimal working temperature of the gas sensitive materials, based on various WO_3_/SnO_2_ HNFs with acetone as the target gas was first determined with pure SnO_2_ HNFs included for comparison. Figure 6 shows that all the four response curves gradually increase when increasing the working temperature from 50°C, and decline at only 170°C for WO_3_/SnO_2_ (0.3%), but 250°C for the other three samples. Additionally, the sensor that is based on WO_3_/SnO_2_ (0.3%) provides the highest response (32) compared with other sensors. Just as expected, WO_3_/SnO_2_ (0.3%) is a promising choice and all the following tests were carried out at the optimal operating temperature of 170°C.

**Figure 7:**
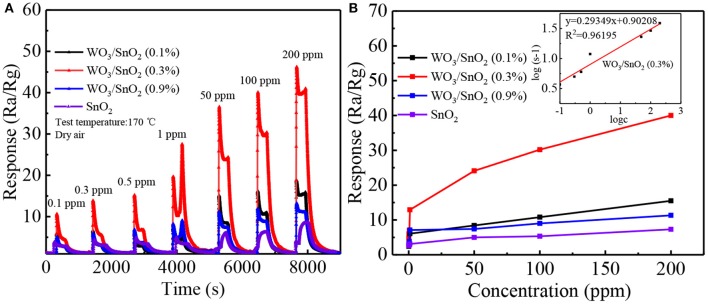
**(A)** Dynamic response transients and **(B)** response curves of the sensors based on above sensing materials as a function of acetone concentration from 0.1 to 200 ppm at 170°C. The fitting curve between concentration and response is represented as inset in **(B)**.

Additionally, four dynamic response change curves of pure SnO_2_ and WO_3_/SnO_2_ sensor devices of different concentrations (0.1–200 ppm) of acetone at 170°C have been provided in Figure 7. As can be seen in Figure 7A, WO_3_/SnO_2_ (0.3%) presents the highest response amplitude compared with the others, as well as the fastest instantaneous response speeds (≤6 s), which proves its favorable response properties. (Figure S3) enlarged response– recovery curve for 100 ppb acetone gas was explored to determine the response/recovery time. At 170°C, for as-fabricated WO_3_/SnO_2_ (0.3%) based sensor toward 100 ppb acetone gas, the response/recovery time was 50 and 200 s, respectively. Furthermore, the sensors display a better fold linear relationship between the response signal vs. acetone concentration (Figure 7B). Such a result clearly indicates that the gas sensitivity of SnO_2_ has been significantly improved through the synergistic effect of the hetero-junction formed by WO_3_ doping, which is also consistent with the results from Figures 4, 5. However, the doping amount of WO_3_ also displays an important role in the synergistic effect, thus affecting the surficial gas catalytical sensing properties.

**Figure 8:**
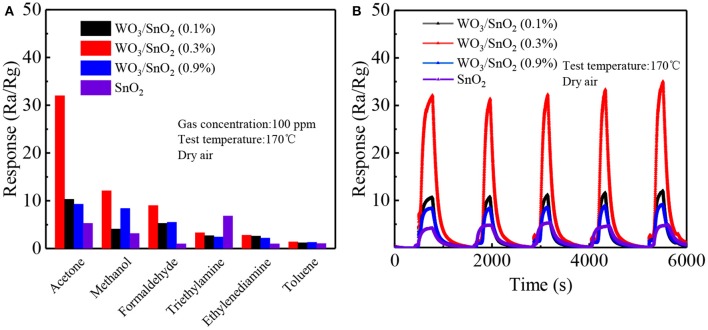
**(A)** The selectivity of the four kinds of sensors toward various industrial gases (100 ppm), and **(B)** the repeatability toward 100 ppm of acetone at 170°C.

In order to further confirm the selectivity of the WO_3_/SnO_2_ (0.3%)-based sensor, 100 ppm of industrial gases such as methanol, formaldehyde, triethylamine, ethylenediamine and toluene were chosen to test at 170°C. Figure 8A shows that the WO_3_/SnO_2_ (0.3%) sample not only exhibits excellent selectivity to acetone gas (32), three times more than other polluting gases, but also the highest response value compared to the other three sensors types. The sensor selectivity was influenced by many factors. On the one hand, the bond dissociation energy of CH_3_-COCH_3_ (352 kJ/mol) is smaller than that of C_2_H_5_O-H, CH_3_O-H (462 kJ/mol), H–COH (368 kJ/mol), H–CH_2_C_6_H_5_ (371 kJ/mol), therefore, acetone is more likely to react with the adsorbed oxygen species than other gas molecules. On the other hand, the polar nature of the surface of the WO_3_/SnO_2_ (0.3 wt%) would accelerate the adsorption of polar molecules. Acetone is much easier to be adsorbed on the surface of WO_3_/SnO_2_ (0.3 wt%) than triethylamine. Consequently, more acetone molecules can react with the adsorbed oxygen species which releases more electrons than any other gas molecule. As a result, the WO_3_/SnO_2_ (0.3 wt%) hetero-nanofibers had a good sensing response and selectivity to acetone gas (Li et al., 2016; Zhang et al., 2017). The more detailed mechanism of adsorption and reaction using DFT calculation and *in situ* instruments, will be discussed in our future work. In Figure 8B, the corresponding desirable repeatability can also be witnessed for the four sensors to 100 ppm of acetone after five circles, especially the most attractive component of WO_3_/SnO_2_ (0.3%). In general, the sensor based on WO_3_/SnO_2_ (0.3%) HNF in this study is a promising candidate for trace indoor or industrial acetone gas sensing.

Last but not the least, to verify the stability of the prepared acetone sensor, the sensitivity of pure SnO_2_ and three groups of WO_3_/SnO_2_ (0.1, 0.3, 0.9 wt%) HNFs were detected every 6 days for 1 month. Figure S2 shows that all the sensors nearly keep their response states, especially the sensor based on WO_3_/SnO_2_ (0.3%) which exhibits quite high and stable response values. Such a result also indicates that the acetone gas sensor has good stability and prospective application.

To give a clear vision on the sensing properties of gas sensors to acetone, comparisons were made in Table 1. As can be seen, the gas sensor based on our WO_3_/SnO_2_ (0.3%) HNF material displays the improved performance in a certain way, such as a lower working temperature of only 170°C. Generally, this work is valuable in enhancing the gas sensing properties by preparation of hollow hetero-structures.

**Table 1:** Sensing performance of various gas sensors toward acetone.

**Materials**	**Method**	**W.T. (^**°**^C)**	**LOD (ppm)**	**Response**	**References**
Ag@CuO-TiO_2_	Hydrothermal	200	1	11	Wang G. et al., 2019
Pt-decorated Fe_2_O_3_ nanocubes	Hydrothermal	139	1	1.8	Zhang et al., 2019a
CoFe_2_O_4_ nanoparticles (SM)	Solvothermal	220	50	16	Zhang H. et al., 2019
Porous NiFe_2_O_4_ microspheres	Hydrothermal	250	0.2	1.2	Zhang et al., 2019b
0.5 wt% Au-ZnO	Sol-geltechnique	172	15	10	Yang M. et al., 2019
BiFeO_3_ nanoparticles	Hydrothermal	350	1	1.8	Chakraborty and Pal, 2019
WO_3_/SnO_2_ heterostructure	Electrospinning	170	0.1	4.7	This work

#### Gas Sensing Mechanism

The basic sensing principle for the n-type semiconductor metal oxides is based on the conductivity changes which are caused by surface gas adsorption and desorption (Wang Q. et al., 2019). In short, oxygen molecules in the air are adsorbed on the metal oxide surfaces. The different oxygen species including O^−^, O^2−^, and O${}_{2}^{-}$ will form at different temperatures by capturing free electrons from the conduction bands of WO_3_ and SnO_2_, e.g., the stable oxygen species on the surface of the sensing material is mainly O^−^ below 300°C (Yamazoe et al., 2003; Rakshit et al., 2012). Thus, the electron concentration will be reduced to form an electron depletion layer, resulting in a higher resistance. When the sensor is exposed to the reduced gas like acetone, it can react with the adsorbed O^−^ and release the captured electrons back, thus decreasing the resistance (Das and Jayaraman, 2014; Yang and Guo, 2016).

**Figure 9:**
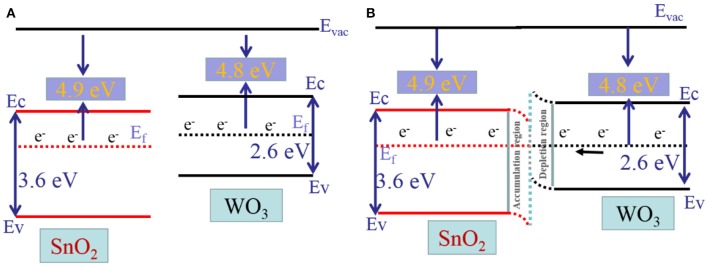
Illustration Diagram of **(A)** the energy band gap of SnO_2_ and WO_3_, **(B)** the energy band gap of the hollow fibrous heterostructure.

On the basis of the above theory, the improved sensing performances for the sensor based on WO_3_/SnO_2_ (0.3%) HNF can be attributed to three factors. First, as shown in Figure 9, the band gap and work function of SnO_2_ is 3.6 and 4.9 eV, while that for WO_3_ it is 2.6 and 4.8 eV, respectively. As the work functions of both are imbalanced, the electrons will transfer from the Fermi level of WO_3_ (higher Fermi level) to that of SnO_2_ (lower Fermi level) until they reach a balance, as displayed in Figures 9A,B. At the equilibrium point, the Schottky barrier with an additional depletion layer between WO_3_ and SnO_2_ are formed, controlling the electron transport efficiency of the heterojunction (Rakshit et al., 2012; Koohestani, 2019). The WO_3_/SnO_2_ hetero-structure provides more electrons to oxygen than pure SnO_2_, then produces more O^−^ species on the materials surface. Therefore, the hetero-structure exhibits better sensing properties (Wal et al., 2009; Lingyue and Shantang, 2018; Yang et al., 2018).

Second, it's also the key factor that the synergetic effect between the interfaces can improve their sensing performances (Gu et al., 2011). It can be concluded from the TEM and XPS results in Figures 4, 5 that, WO_3_ nanoparticles have been incorporated into the SnO_2_ HNFs successfully, thus both of them become highly accessible for the adsorption of oxygen species, add the thickness of the depletion layer at the interfaces and join in the reaction with acetone to a greater extent. This promotes the sensing response but helps reduce the working temperature of pure SnO_2_ (Parthibavarman et al., 2018; Wang L. et al., 2019). However, the heterojunctions with different contents of WO_3_ showed different gas sensing properties during the test, which also indicates that the optimal proportion of dopant is quite important in the construction of heterojunctions (Stojadinović et al., 2016).

The last factor has a lot to do with the morphology of the heterojunction. Many advantages exist in the HNFs produced by the electro-spinning method, since compared to the other three dimensions, the first characteristic of 1D nanostructure is its smaller dimension structure and high aspect ratio, which could efficiently transport electrical carriers along one controllable direction, making it highly suitable for moving charges in integrated nanoscale systems (Wal et al., 2009; Gu et al., 2011; Lu et al., 2011; Yuan et al., 2011; Wang et al., 2016). Additionally, the grain size also greatly affects the gas sensitivity. The region within a Debye length of the surface is known as the depletion region because it is depleted of its normal charge carriers. The Debye length may change to more or less when oxygen is adsorbed on the surface, which in turn causes a measurable change in the resistance. It has been overwhelmingly shown that when crystallite sizes are below about 20 nm, sensor response drastically increases (Miller et al., 2014), similar to the explanation presented in the XRD part, where the grain size calculations of various heterojunctions at 17.53, 15.71, and 20.56 nm in the WO_3_/SnO_2_ (0.1%,0.3%,0.9%) composite, and the smallest grain size of 15.71 nm in the WO_3_/SnO_2_ (0.3%), contributes to its best gas sensitivity among all the samples. The gas sensitivity was not controlled by the grain size of the main phase alone, but also the particle size, density, and distribution of the doped catalyst WO_3_. As the doping amount of WO_3_ is 0.1 wt%, small WO_3_ nanoparticles are sparsely dispersed in SnO_2_ nanotubes with a lower density and less heterogeneous nodes. The increased charge carrier density would have little impact on depletion regions and the overall electrical conduction in the WO_3_/SnO_2_ heterostructure. When the doping of WO_3_ is higher than 0.3 wt%, the crystallization of WO_3_ is accompanied by the growth of SnO_2_ crystallite size and the loss in specific surface area, leading to a less effective target gas diffusion, less reduction of depletion regions and deteriorated sensor response. Motivated by such facts, when the content of WO_3_ is above 0.3 wt% results in a weakened gas response.

## Conclusion

The heterogeneous structure and pure SnO_2_ of three components of WO_3_/SnO_2_ hollow nanofibers (HNF) were prepared by electro spinning in this study. The grain size measured by TEM and calculated by scherer formula also proved that the group with the best gas sensitivity was WO_3_/SnO_2_ (0.3%) HNF. Moreover, the large specific surface area characteristic of the hollow structure and the enhanced surface activity as the grain size decreases to the nanometer level providing a good way for the gas to enter the semiconductor material. The sample of the WO_3_/SnO_2_ (0.3%) component has the best selectivity for acetone in several representative industrial waste gases at the optimal operating temperature of 170°C. In particular, while the acetone concentration is as low as 100 ppb a good response was shown as well (4.7), and the response/recovery time was 50/200 s, respectively. This research can provide significant references for acetone gas sensors with low temperature and low detection limits.

## Data Availability Statement

All datasets generated for this study are included in the article/Supplementary Material.

## Author Contributions

HS initiated the experiments and draft. MH, HF, and SW helped prepare the samples. LW, JL, and KY contributed equally to this work, and they designed and completed the manuscript. YW offered useful comments on the technical methods.

### Conflict of Interest

The authors declare that the research was conducted in the absence of any commercial or financial relationships that could be construed as a potential conflict of interest.
