# Supplementary material for: Hollow WO3/SnO2 Hetero-Nanofibers: Controlled Synthesis and High Efficiency of Acetone Vapor Detection
Source: Front Chem. 2019 Nov 19;7:785. doi: 10.3389/fchem.2019.00785 (PMC6884032; doi:10.3389/fchem.2019.00785)
Supplement: Supplementary file 1 [file Table_1.docx]

Supplementary Material

**Hollow** WO_3_/SnO_2_ **hetero-nanofibers: Controlled synthesis and high efficiency of acetone vapor detection**

**Hongyun Shao^a,b^, Minxuan Huang^a,b^, Hao Fu^a,b^, Shaopeng Wang^a,b,c^, Liwei Wang^a,b,c,^*, Jie Lu^d,^*, Yinghui Wang^a,b^, Kefu Yu^a,b,^***

^a^ School of Marine Sciences, Guangxi University, Nanning, 530004, China

^b^ Guangxi Laboratory on the Study of Coral Reefs in the South China Sea, Nanning, 530003, China

^c^ Guangxi Key Laboratory of Processing for Nonferrous Metallic and Featured Materials, Nanning, 530003, China

^d^ College of Life Science and Technology, Guangxi University, Nanning, 530004, China

***Corresponding Authors**

Email: wangliwei0427@163.com; jlu92@163.com; kefuyu@scsio.ac.cn;

**Supplementary Material available:** The following files are available free of charge.

**Fig. S1.** EDS spectrum image of 0.3 wt% WO_3_/SnO_2_ HNFs.

**Fig. S2.** Long-term stability of the sensors consisting of WO_3_/SnO_2_ HNFs to 100 ppm of acetone within 30 days.

**Fig. S3.** Response/recovery times of 0.3 wt% WO_3_/SnO_2_ sample to 100 ppb acetone.


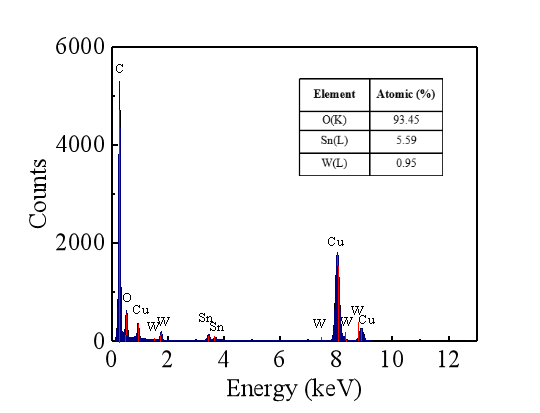


**Fig. S1.** EDS spectrum image of 0.3 wt% WO_3_/SnO_2_ HNFs.

**Fig. S2.** Long-term stability of the sensors consisting of pure SnO_2_ and three groups of WO_3_/SnO_2_ (WO_3_ of 0.1, 0.3, 0.9 wt%) to 100 ppm acetone within 30 days.

**Fig. S3.** Response/recovery times of 0.3 wt% WO_3_/SnO_2_ sample to 100 ppb acetone.
